# P-758. Single-Dose Long-acting Lipoglycopeptide Clinical Pathway for Treatment of Cellulitis in the Emergency Department: A How-to Guide Based on Real-World Implementation at a Community Hospital Health System

**DOI:** 10.1093/ofid/ofaf695.969

**Published:** 2026-01-11

**Authors:** Cole Orlikowski, Nicholas Torney, Aleah Hunt, William Britton, Cynthia Nichols

**Affiliations:** Henry Ford Hospital, Detroit, MI; Munson Medical Center, Traverse City, MI; Munson Medical Center, Traverse City, MI; Munson Medical Center, Traverse City, MI; Munson Medical Center, Traverse City, MI

## Abstract

**Background:**

Long-acting lipoglycopeptides (LaLGPs) are emerging as promising alternatives for the treatment of cellulitis in patients who might otherwise require short-stay hospitalization. Compared to standard intravenous antibiotic regimens, LaLGPs may offer advantages in both clinical efficiency and healthcare resource utilization. In September 2024, Munson Medical Center (Traverse City, MI) implemented a LaLGP-based clinical pathway in the emergency department (ED) to streamline cellulitis care and reduce hospital admissions.

**Methods:**

This single-center retrospective cohort study compared two groups: a pre-implementation cohort (patients hospitalized ≤ 72 hours for cellulitis from July 2022 – August 2024) and a post-implementation cohort (patients receiving ED-administered dalbavancin from September 2024 – April 2025). The primary outcome was 30-day hospital admission for recurrent cellulitis. Secondary outcomes included ED or urgent care visit within 30 days for recurrent cellulitis, antibiotic prescriptions for recurrent cellulitis within 30 days, and all-cause hospital admission within 72 hours. A cost analysis estimated potential savings by calculating avoidable hospital days using publicly available financial data and the average length of stay from the pre-implementation cohort.

**Results:**

Of the 208 patients screened, 60 met inclusion criteria: 48 in the pre-implementation cohort and 12 in the post-implementation cohort. No statistically significant differences were observed between cohorts in 30-day outcomes for recurrent cellulitis, including hospital admissions (2.1% vs. 8.3%; p=0.36), ED or urgent care visits (4.2% vs. 8.3%; p=0.50), antibiotic prescriptions (27% vs. 8.3%; p=0.26), or 72-hour all-cause hospital admission (0% vs. 8.3%; p=0.20). Over the 8-month study period, an estimated 22.8 hospital days were avoided in the ED-LaLGP cohort (n=12). Excluding initial ED visit costs, this corresponds to a projected cost savings of $52,919 USD – approximately $4,409 USD per patient.

**Conclusion:**

These findings support the use of an ED-LaLGP clinical pathway as a viable strategy to reduce short-stay hospitalizations for cellulitis, offering comparable clinical outcomes with improved cost-efficiency for both patients and healthcare systems.

**Disclosures:**

All Authors: No reported disclosures

